# Wine secondary aroma: understanding yeast production of higher alcohols

**DOI:** 10.1111/1751-7915.12770

**Published:** 2017-07-11

**Authors:** Ramon Gonzalez, Pilar Morales

**Affiliations:** ^1^ Instituto de Ciencias de la Vid y del Vino (CSIC‐ Universidad de La Rioja‐ Gobierno de La Rioja) Logroño Spain

Contribution of yeasts to the sensory attributes of fermented foods and beverages goes far beyond sugar consumption and ethanol and carbon dioxide production. It includes some major by‐products of fermentation, like glycerol or acetic acid, and hundreds of aroma‐active compounds, including higher alcohols, esters, aldehydes, organic acids, volatile fatty acids or carbonyl compounds, as main constituents of the secondary or fermentation aroma of grape wine (Styger *et al*., 2011). In addition, they contribute to the enzymatic transformation of some neutral precursors, originating from the substrate, into odour‐active molecules, so enhancing primary or varietal aroma in wine.

The structural relationship between some higher alcohols, esters, organic acids and amino acids (which contribute some of the nitrogen sources in natural fermentation substrates) suggests the later might be the precursors of some important constituents of the secondary aroma. For example, isobutyl alcohol, active amyl alcohol and isoamyl alcohol are structurally related to valine, isoleucine and leucine respectively (Lambrechts and Pretorius, 2000), and they are linked through the Ehrlich pathway (Hazelwood *et al*., 2008). Although a contribution of amino acid carbon chains to the production of these higher alcohols has been clearly established (Reazin *et al*., 1970, 1973), there are also clear‐cut evidences that the input from yeast central carbon metabolism is by no means negligible, being in most cases the main origin of these branched‐chain alcohols (Rankine, 1967; Reazin *et al*., 1970).

The topic of the dependence of higher alcohol production (and the relative contribution of each metabolic pathway) on the nitrogen content of the fermentation substrate has generated a wealth of scientific literature. However, drawing general rules has proven difficult, with results from different studies pointing in opposite directions. This suggests complex non‐linear relationships between the availability of amino acids or nitrogen sources in general, and the release of aroma compounds by *Saccharomyces cerevisiae*. An illustration of complex dependences between aroma‐active compounds and amino acid availability was shown by Hernández‐Orte *et al*. (2002).

One intuitive idea about amino acid metabolism during fermentation, and more specifically in winemaking, is that, once uptaken by yeast, amino acids whose availability is below biosynthetic requirements, will be directed to protein synthesis (rather than being used for other purposes). Indeed, in order to avoid ‘in silico’ futile cycles, this assumption has been used in metabolic flux analysis in order to choose between anabolic or catabolic reactions to be incorporated in the metabolic network for each amino acid (Quirós *et al*., 2013). Early this year researchers from INRA at Montpellier published an elegant work based on stable isotope tracers, with an exhaustive analysis of the fate of amino acids in laboratory fermentation trials (Crépin *et al*., 2017). According to their results, only a small proportion of the amino acids from synthetic must is directly incorporated into proteins by *S. cerevisiae*. Most of them are broken down by transamination and the amino groups used for *de novo* synthesis of proteinogenic amino acids. The level of transamination is roughly independent of amino acid availability or anabolic requirements. In agreement with other authors, and despite initial breakdown of amino acids is in the origin of the release of some higher alcohols, this is a minor contribution to the total amount finally produced. Central carbon metabolism plays a key role in the formation of these metabolites. However, not all of them strictly follow the above described general rules (Crépin *et al*., 2017).

In this issue, Rollero *et al*. (2017, this issue) used ^13^C‐labelled leucine and valine, and introduce variations in synthetic must composition, to address the old question of the influence of nitrogen sources (and lipids) on the production of fermentative aromas. They confirm the relatively low direct incorporation of exogenous amino acids into proteins under all environmental conditions, and the overall low contribution of amino acid carbon backbones to global higher alcohol production. They also describe dependence of these contributions on the growth phase, and how the flux distribution around the α‐ketoisovalerate and α‐ketoisocaproate nodes is affected by nitrogen and phytosterol availability. Interestingly, their detailed analysis is providing mechanistic explanations to phenomena like the maximal level of higher alcohol production found at intermediary nitrogen doses (Rollero *et al*., 2017, this isssue), which take into account the contribution of amino acid catabolism and central carbon metabolism to the α‐ketoacids pool, and their dependence on nitrogen source availability. Also, patterns of isotope enrichment confirm some higher alcohols (isobutanol and isoamyl alcohol), esters (isoamyl acetate and isobutyl acetate) and organic acids (isovalerate and isobutyrate), structurally related to valine and leucine, all derive from the same α‐ketoacids.

Until recently, our understanding of yeast regulation of the metabolism of nitrogen sources, and the production of volatile compounds were either based on relatively simple synthetic media, or confounded by contradictory results from complex or natural media. This work (Rollero *et al*., 2017, this issue) opens an avenue of possibilities to improve our understanding of yeast metabolic features that are relevant for the quality of wine and all other food and beverages made with *S. cerevisiae* as the main fermentation agent. Similar approaches would eventually allow getting insight into the response to industrially relevant variables of metabolic pathways involved in the production of other important aroma compounds, including for example other higher alcohols and esters (e.g. phenylethyl alcohol and phenylethyl acetate), or sulfur‐containing aroma compounds.

In addition, there is increasing awareness on the contribution of non‐*Saccharomyces* yeast species to wine sensory features, either as wild microbiota, or as complementary starters in simultaneous or sequential inoculation. In most cases, both the metabolic pathways leading to aroma‐active compounds in these alternative yeast species, and their regulation, are, at most, poorly known. Very often we must resort to parallelism with *S. cerevisiae* in order to interpret experimental observations. The use of isotope tracers would certainly help getting quick insight into relevant metabolic features of *Torulaspora delbrueckii*,* Lachancea thermotolerans* or *Metschnikowia pulcherrima*, among other yeast species. Together with research on *S. cerevisiae*, this would help developing winemaking strategies aimed to the desired wine styles, or to solve specific problems in the winemaking industry.

## Conflict of interest

The authors declare no conflict of interest.
